# Assessing the impact of enteral nutrition on peripancreatic exudate in acute pancreatitis using CT volume measurement

**DOI:** 10.3389/fmed.2025.1660818

**Published:** 2025-09-18

**Authors:** Cui Hu, Chengcheng Tian, Chen Chen, Mengxue Huang, Jun Li, Xin Gao, Dandan Du, Jiren Wang, Lixue Zhang, Yu Wang, Mengqi Huang, Nannan Zhu, Huizhong Gan, Hao Ding, Qiao Mei

**Affiliations:** ^1^Department of Gastroenterology, The First Affiliated Hospital of Anhui Medical University, Hefei, China; ^2^Department of Gastroenterology, Jinzhai County People’s Hospital, Lu’an, China; ^3^Department of Gastroenterology, Taihe County People’s Hospital, Fuyang, China; ^4^Department of Radiology, The First Affiliated Hospital of Anhui Medical University, Hefei, China; ^5^Department of Gastroenterology, The Third Affiliated Hospital of Anhui Medical University, Hefei, China

**Keywords:** acute pancreatitis, local complications, enteral nutrition, peripancreatic, exudate volume, computed tomography

## Abstract

To investigate the effect of enteral nutrition (EN) on peripancreatic exudate in MSAP and SAP patients using CT volume measurement. This retrospective study included 328 patients diagnosed with MSAP and SAP from October 2017 to June 2023. Patients were divided into EN and non-EN groups and further stratified into acute peripancreatic fluid collection (APFC) and acute necrotic collection (ANC) groups based on local complications. Abdominal CT images obtained within 24 h of admission and within 15 days after therapy were analyzed using Itk-Snap software to outline and calculate peripancreatic exudate volumes. Volume differences before and after therapy were compared between groups. A 1 year follow-up was conducted to compare the time of complete resolution of peripancreatic exudate between groups. After EN therapy, patients have significantly less peripancreatic exudate than those who do not receive EN. In the APFC and ANC groups, EN was associated with a greater reduction in exudate than that in the non-EN group. The time to complete resolution of exudate was shorter in the EN group than in the non-EN group. EN promotes the absorption of peripancreatic exudate and improves the prognosis of patients with MSAP and SAP.

## 1 Introduction

Acute pancreatitis (AP) is a complex and varied disease with an overall increase in incidence of 3.07% per year (1). A total of 80% of patients develop mild acute pancreatitis (MAP), which can be cured by fluid resuscitation, nutritional support therapy and other therapies (2, 3). The remaining 20% of severely ill patients are characterized by pancreatic necrosis or distal organ failure and are expected to require intensive care and possible surgical intervention, with mortality rates up to 20% (3, 4).

In AP, a large number of digestive enzymes are activated within the pancreas, leading to autodigestion of the pancreatic tissue. Concurrently, there is significant leakage of digestive fluids, causing damage to surrounding tissues. This process triggers overactivation of leukocytes and a cascade of inflammatory factors, which can result in extensive endothelial cell damage, capillary leak syndrome and microcirculatory disturbances, leading to systemic inflammatory response syndrome (SIRS) responses and multiple organ dysfunction syndrome (MODS). In patients with moderately severe acute pancreatitis (MSAP) and severe acute pancreatitis (SAP), the leakage of digestive fluids and plasma infiltration into the tissue space leads to increased abdominal exudation. This can further progress to complications such as local pancreatic and peripancreatic necrosis, abdominal or retroperitoneal hemorrhage, intestinal failure, and splenic vein thrombosis. Therefore, promoting the rapid absorption of peritoneal exudation during the therapy of AP may alleviate local complications, shorten the course of the disease and improve the prognosis.

AP is ultimately an inflammatory process in which high levels of inflammation induce a hypermetabolic state, leading to increased fat and protein catabolism, insulin resistance and weight loss, and placing patients with severe disease at moderate to high nutritional risk (5, 6). Therefore, nutritional support is one of the cornerstones of management (7). Enteral nutrition (EN) is believed to preserve the integrity of the gut mucosa, stimulate intestinal motility, prevent bacterial overgrowth, and increase the splanchnic blood flow (5). Enteral feeding also prevents the translocation of bacteria that seed pancreatic necrosis (8, 9). EN in patients with MSAP and SAP seems to prevent infectious complications (10). Several randomized controlled trials and systematic reviews/meta-analyses have clearly demonstrated that, in patients with SAP, EN is safer and better tolerated than parenteral nutrition, with significant reductions in complication, multi-organ failure, and mortality rates (11–13).

For MAP, nutritional support is usually not required. Once pain has diminished along with nausea and vomiting, oral nutrition can be started. Patients with MSAP and SAP at risk for necrosis should be started on EN as soon as possible, as there is a clear mortality benefit for those who receive early enteral feeding (14). For MSAP and SAP patients, the guidelines recommend that EN can be started within three days of onset (6). While EN has demonstrated significant benefits in improving patients’ systemic nutritional status, reducing infection risk, and lowering mortality rates, clinical observations suggest that EN may accelerate the resolution of peripancreatic exudate. However, there remains a lack of systematic research directly examining the effect of EN on local complications associated with AP. Therefore, it is essential to develop reliable methods to evaluate the effects of EN on these local complications.

The assessment of AP is mostly indirect, relying on biomarkers such as C-reactive protein (CRP), and procalcitonin (PCT), as well as scoring systems such as the Ranson score, the Acute Physiology and Chronic Health Evaluation II (APACHE II) score, and the Bedside Index for Severity in Acute Pancreatitis (BISAP) score. However, these methods do not provide a direct and accurate assessment of peripancreatic exudate. In imaging assessment, CT is not only used to diagnose AP but also to assess its severity and identify complications such as necrosis (infected or not), pseudocyst formation, and vascular or extra-pancreatic complications (15). Although the modified computed tomography severity index (MCTSI) is currently widely used and may accurately diagnose the extent and severity of pancreatitis (16), it lacks accurate measurement of pancreatic exudate volume. The purpose of this study was to directly and quantitatively evaluate the changes in peripancreatic exudate before and after EN therapy using a new method with Itk-Snap software to delineate the peripancreatic exudate boundary and calculate its volume, thereby determining the effect of EN therapy on peripancreatic exudate in patients with MSAP and SAP.

The significance of this innovative method is that it can provide clinicians with a more accurate means to evaluate pancreatic exudation and compensate for the shortcomings of traditional imaging scores in quantitative analysis. Additionally, by quantitatively assessing the changes in peripancreatic exudate, this method offers direct imaging evidence of the efficacy of EN therapy.

## 2 Materials and methods

### 2.1 Study design and participants

Eligible patients with MSAP and SAP admitted to our institution between October 2017 and June 2023 were included in this retrospective study. This study was approved by the Institutional Review Board and Ethics Committee of the First Affiliated Hospital of Anhui Medical University (ChiCTR2400090429). The Ethics Committee approved the exemption from informed consent. Demographic characteristics (e.g., age, gender, BMI, and history of diabetes mellitus) were retrieved from the electronic health records.

AP was diagnosed according to the International Consensus Diagnostic Criteria of 2012. Patients with MSAP and SAP were classified according to the Revised Atlanta Classification (RAC) as follows: (1) MAP, with no local complications or organ failure; (2) MSAP, with transient organ failure (< 48 h), local complications and/or exacerbation of comorbidities; and (3) SAP, with persistent organ failure (≥ 48 h), most often respiratory, with or without local complications (17).

Early complications of MSAP and SAP include acute peripancreatic fluid collection (APFC) and acute necrotic collection (ANC). In this study, all patients underwent at least one enhanced CT examination during their hospitalization. According to the results of enhanced CT examination, the patients were divided into the APFC group and the ANC group, and the MCTSI score was conducted simultaneously.

The exclusion criteria included the following: (1) patients with MAP meeting the RAC criteria, (2) patients transferred from other hospitals, (3) patients undergoing one or more peritoneal puncture drainages due to peripancreatic effusion, (4) patients with pancreatic infected necrosis, and (5) patients who received EN for less than 4 weeks.

Patients were divided into two groups based on whether they received EN after admission: the enteral nutrition group (EN group) and the non-enteral nutrition group (non-EN group). Both groups received standardized and appropriate fluid therapy upon admission. Specifically, during fluid therapy, crystalloids, primarily balanced fluids, were administered at a rate of 5–10 ml/kg/h during the first 12–24 h. The infusion rate was further titrated based on clinical and biochemical targets of perfusion. The clinical targets included a heart rate of less than 120/min, a mean arterial pressure between 65 and 85 mmHg, and a urinary output of more than 0.5 ml/kg/h. The biochemical targets included a hematocrit of 35%–45% and a blood urea nitrogen concentration (18).

Patients in the EN group underwent EN therapy initiation within 1–3 days of admission based on the patient’s symptoms via a nasojejunal tube, which was placed either endoscopically or radiologically. After tube placement, nasoenteric feeding was started at 20 ml/h for the first 24 h, with careful monitoring of abdominal and gastrointestinal symptoms. Subsequently, the feeding rate was increased to 45 ml/h after 24 h, to 65 ml/h after 48 h, and to full nutrition after 72 h, based on the patient’s body weight. Full nutrition was defined as an energy target of 25 kcal/kg/day for patients in the intensive care unit (ICU) and 30 kcal/kg/day for patients in the ward (19). All patients received EN in the form of a whole protein-based formula. Patients could consume water and a small amount of a low-fat diet through the mouth while receiving EN without intravenous fluid replacement to avoid interference. In contrast, patients in the non-EN group did not receive EN during their hospital stay. Instead, they consumed food orally.

**FIGURE 1:**
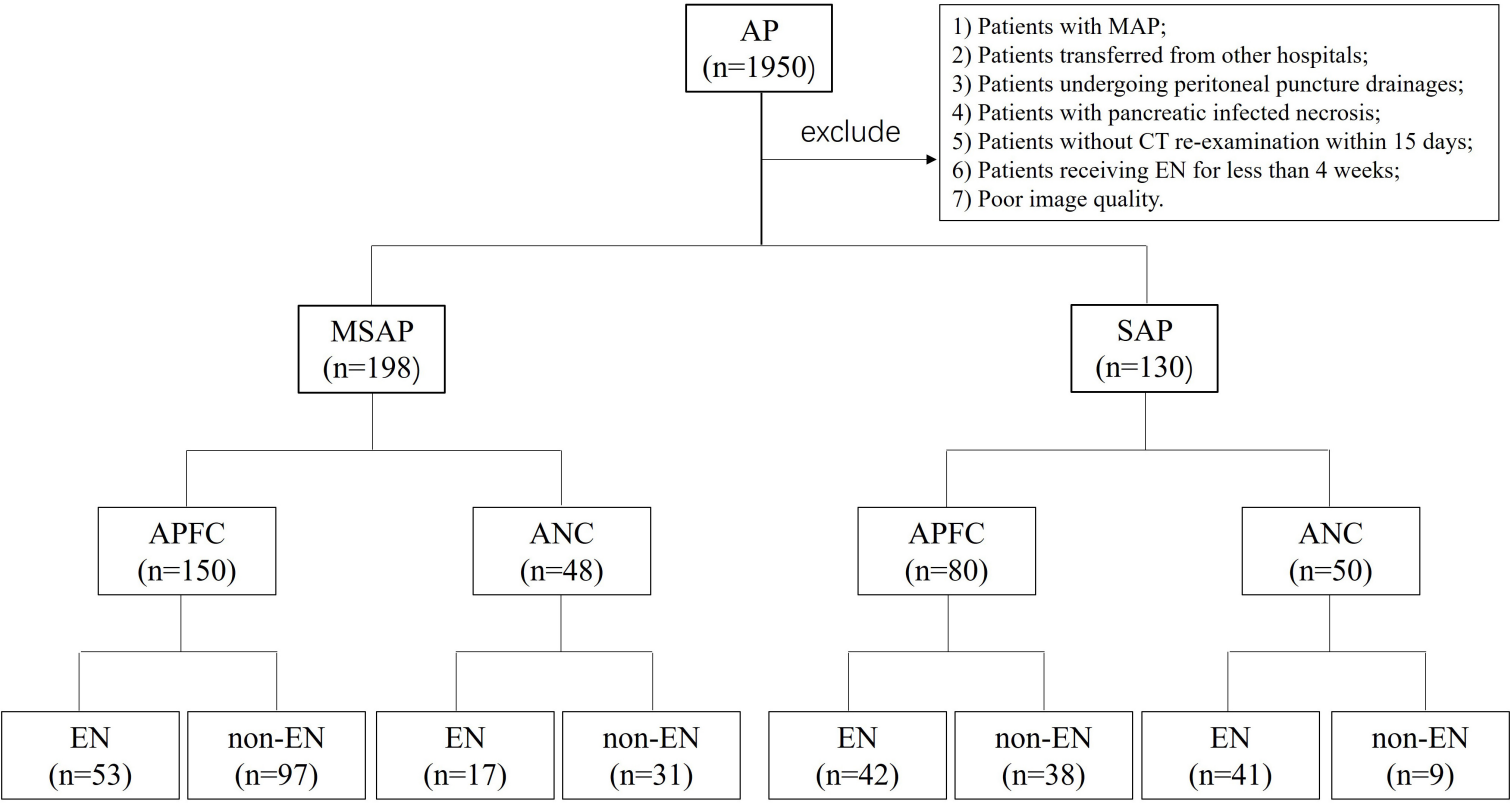
Patient screening and study roadmap. AP, acute pancreatitis; MSAP, moderately severe acute pancreatitis; SAP, severe acute pancreatitis; APFC, acute peripancreatic fluid collection; ANC, acute necrotic collection; EN, enteral nutrition; non-EN, non-enteral nutrition.

For all patients, the first abdominal CT scan was performed within 24 h of admission, and the second abdominal CT scan was conducted within 15 days of admission. Patients with poor CT image quality were excluded. Given that EN therapy typically began within 1–3 days of admission, the time interval between the first CT scan and the initiation of EN was not considered in this study. The patient screening process and research roadmap are illustrated in Figure 1.

### 2.2 Image acquisition and processing

**FIGURE 2:**
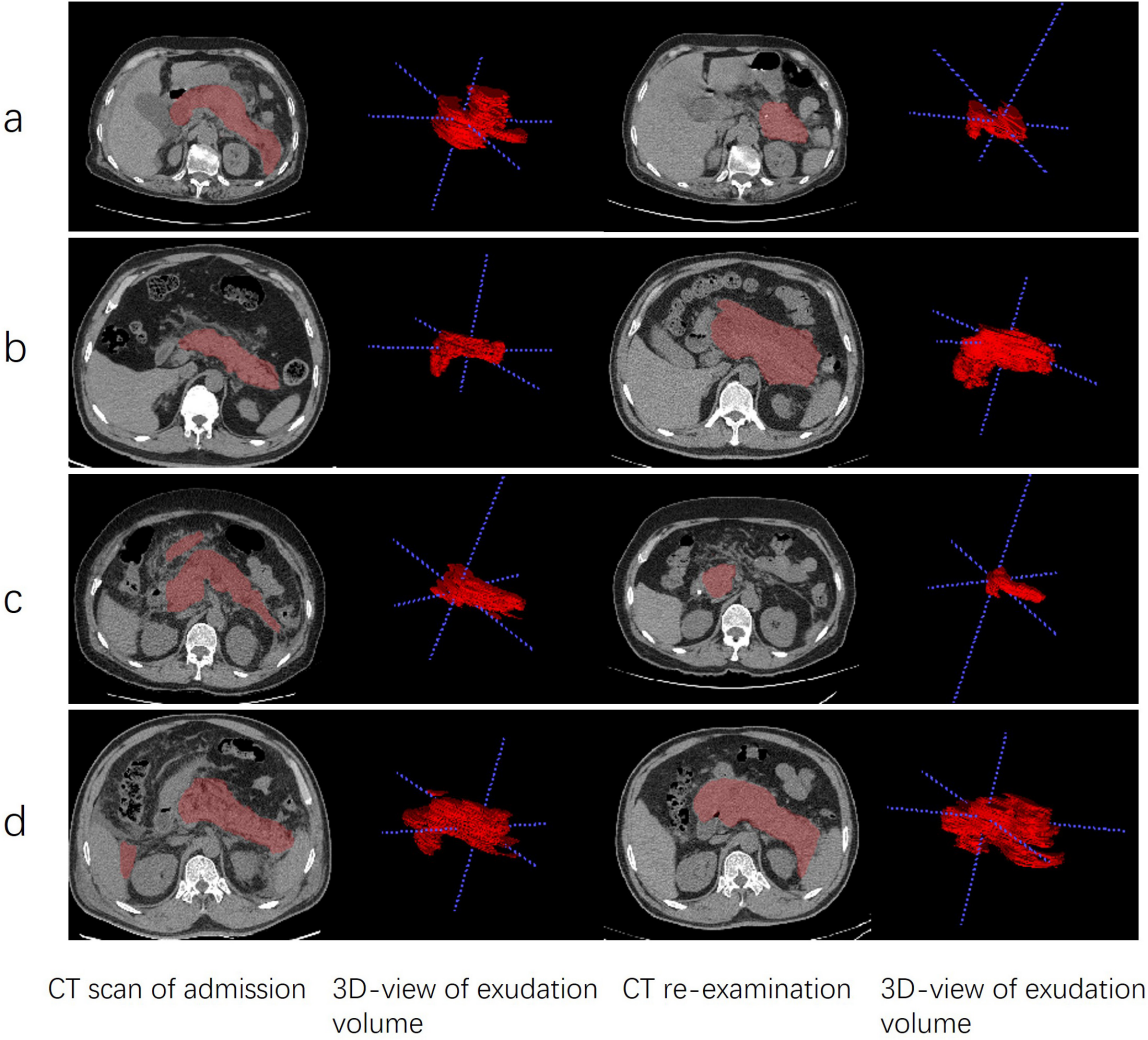
Comparison of AFPC and ANC patients before and after EN and non-EN. **(a)** EN therapy in a 75 years-old female patient with AFPC. **(b)** non-EN therapy in a 55 years-old male patient with AFPC. **(c)** EN therapy in a 43 years-old male patient with ANC. **(d)** non-EN therapy in a 50 years-old male patient with ANC. Each pair of rows represents the same axial section. The 3D view of the exudate volume corresponds to the picture on the left. APFC, acute peripancreatic fluid collection; ANC, acute necrotic collection; EN, enteral nutrition; non-EN, non-enteral nutrition.

Two non-contrast CT scans were performed for each patient with a slice thickness of 1.25 mm, covering the anatomical range from the diaphragm to the pubic symphysis. The acquired images were saved in DICOM format, a standard widely adopted in medical imaging that ensures the preservation of comprehensive image metadata. These DICOM-formatted CT images were subsequently imported into Itk-Snap software (version 3.8, America). Using the Itk-Snap tagging tool, the pancreatic area and surrounding exudate in the abdominal cavity were manually delineated layer by layer. This meticulous boundary delineation process ensured the accuracy of the segmentation results, thereby laying a solid foundation for subsequent volume calculations. All segmentations were performed by trained doctors and later reviewed and corrected by two senior radiologists when needed. The volume calculation tool integrated within Itk-Snap was used to measure the volume of peripancreatic exudate in each individual 2D-axial slice. Subsequently, the volumes of the pancreas and peripancreatic exudate from all 2D axial slices were summed to obtain the total 3D exudate volume. This tool automatically counts the voxels within the segmented region and calculates the volume based on the voxel spacing specified in the image metadata. The volume difference between two CT scans was calculated by subtracting the volume of peripancreatic exudate in the CT images before therapy from the volume of the CT images after therapy. The results of image processing are shown in Figure 2.

### 2.3 Efficacy evaluation indicators for peripancreatic exudate

The changes in peripancreatic exudate volume in two CT scans were compared between the EN and non-EN groups. Additionally, the changes in peripancreatic exudate volume in two CT scans were compared between patients with or without EN at the early stage with local complications, and the therapeutic effect of EN on local complications was evaluated. The difference in peripancreatic exudation volume between EN and non-EN patients at different CT re-examination intervals was compared. All patients received EN for at least one month. Depending on the medical condition of the patients, some required continued treatment for up to three months. One month after discharge, all patients underwent abdominal CT examinations. Subsequently, based on the examination results, CT examinations were conducted every few months until the exudation resolved. The patients were followed up for one year after discharge. The time of complete resolution of peripancreatic exudate in the two groups was recorded and compared.

### 2.4 Statistical analysis

Quantitative variables with a normal distribution are expressed as means ± standard deviations (Mean ± SD). In cases where the data exhibit a non-normal distribution, the median and interquartile range [Median (IQR)] are utilized for data representation. For comparison between two independent groups, independent sample Student’s *t*-test was used for continuous variables with normal distribution, and Mann-Whitney U test was used for variables with non-normal distribution. Counting data are expressed as frequencies (*n*) and percentages (%). Counting data were analyzed via the Pearson chi-square test and Fisher’s exact test. All data and statistical analyses were performed using SPSS version 26.0 (IBM SPSS Statistics). In this study, the Kaplan-Meier (KM) curve was employed to analyze the follow-up outcomes. KM curve was performed by R version 4.4.1 (R Project for Statistical Computing). A two-tailed *P* < 0.05 was considered statistically significant.

## 3 Results

### 3.1 Patient baseline characteristics

A total of 328 patients with MSAP and SAP hospitalized in our hospital from October 2017 to June 2023 were included in this study, including 153 patients (88 males) in the EN group and 175 patients (110 males) in the non-EN group. There were no statistically significant differences between the two groups of patients with respect to age, gender, BMI, and other factors. However, there were statistically significant differences in disease severity, BISAP scores within 24 h of admission and length of hospital stay (Table 1).

**TABLE 1:** Patient baseline characteristics.

Characteristic	EN group (*n* = 153)	Non-EN group (*n* = 175)	*P*-value^a^
**Gender**			0.324
Male	88 (57.52)	110 (62.86)	–
Female	65 (42.48)	65 (37.14)	–
**Age, y**			0.311
Median (IQR)	45 (34–58.5)	48 (34–63)	
**BMI, kg/m^2^**	25.972 ± 3.975	25.444 ± 4.072	0.237
**Smoking history**			0.796
Yes	27 (17.65)	29 (16.57)	–
No	126 (82.35)	146 (83.43)	–
**Alcohol consumption history**			0.064
Yes	39 (25.49)	30 (17.14)	–
No	114 (74.51)	145 (82.86)	–
**History of diabetes mellitus**			0.589
Yes	27 (17.65)	27 (15.43)	–
No	126 (82.35)	148 (84.57)	–
**Etiology of AP**			0.440
Gallstones	63 (41.18)	76 (43.43)	–
Hypertriglyceridemia	63 (41.18)	61 (34.86)	–
Other^b^	27 (17.65)	38 (21.71)	–
**SIRS at admission**			0.053
Yes	105 (68.63)	102 (58.29)	–
No	48 (31.37)	73 (41.71)	–
**BISAP within 24 h after admission**			0.049
Median (IQR)	2 (1–2)	2 (1–2)	–
**RAC**			< 0.001
Moderate	70 (45.75)	128 (73.14)	–
Severe	83 (54.25)	47 (26.86)	–
**MCTSI score**			0.003
Median (IQR)	6 (4–8)	6 (4–6)	–
**Length of stay, d**			< 0.001
Median (IQR)	16 (13–20)	13 (10–17)	–

*^a^*Student’s *t*-test, Pearson Chi-squared test, Mann-Whitney U test.

*^b^*Alcoholic, post-ERCP, malignant tumor, intraductal papillary mucinous neoplasm and idiopathic pancreatitis. EN, enteral nutrition; non-EN, non-enteral nutrition. RAC, the Revised Atlanta Classification.

In this study, the EN group exhibited a higher proportion of severe cases. The placement of an EN tube is an interventional procedure, which may induce discomfort and is perceived as invasive. For patients with MSAP, these factors often result in a reluctance to undergo EN support. Given the retrospective nature of the study, the EN group was overrepresented by patients with more severe conditions at the time of enrollment. The BISAP score within 24 h of admission and MCTSI score was consistent with the severity of the patient’s disease. The general data of patients in the EN and non-EN groups were further compared according to disease severity classification, as shown in Supplementary Tables 1, 2.

### 3.2 Comparison of peripancreatic exudate volume

As shown in Table 2, in both MSAP and SAP patients, the volume of peripancreatic exudate decreased significantly in the EN group after therapy compared with before therapy. In contrast, the volume of peripancreatic exudate increased in the non-EN group compared with that before therapy. The difference in peripancreatic exudate volume between the two groups was statistically significant.

**TABLE 2:** Comparison of peripancreatic exudate volume difference between the EN and non-EN groups (× 10^4^ mm^3^).

RAC	EN group	Non-EN group	*P*-value
MSAP	*n* = 70	*n* = 128	< 0.001
	−15.670 (−29.413∼−1.370)	2.370 (−5.025∼11.728)	
SAP	*n* = 83	*n* = 47	< 0.001
	−7.380 (−31.850∼2.090)	10.890 (0.150∼26.330)	
MSAP + SAP	*n* = 153	*n* = 175	< 0.001
	−12.280 (−30.790∼0.090)	3.440 (−4.330∼15.270)	

EN, enteral nutrition; non-EN, non-enteral nutrition. MSAP, moderately severe acute pancreatitis; RAC, the Revised Atlanta Classification; SAP, severe acute pancreatitis.

In Table 3, in both MSAP and SAP patients, the volume of peripancreatic exudate in APFC patients treated with EN was significantly reduced after therapy, whereas in APFC patients without EN, the volume of peripancreatic exudate increased. These findings indicate that EN therapy is associated with a reduction in peripancreatic exudate in APFC patients. Similarly, in ANC patients treated with EN, the volume of peripancreatic exudate was significantly decreased after therapy, while in those without EN, the volume increased. This finding further supports the role of EN in reducing peripancreatic exudate in ANC patients. As shown in Table 4, in patients with APFC, the volume of peripancreatic exudate at re-examination CT scans within ≤ 7 days and between 8 and 15 days was significantly lower in those treated with EN compared with those without EN. This indicates that EN can promote peripancreatic exudate absorption in APFC patients starting from the first week of treatment. In patients with ANC, the volume of peripancreatic exudate at re-examination CT scans between 8 and 15 days was significantly lower in MSAP and SAP patients treated with EN compared with those without EN. However, among MSAP and SAP patients, those treated with EN did not show a statistically significant difference in the change in peripancreatic exudate volume at re-examination CT scans within ≤ 7 days compared with those without EN.

**TABLE 3:** Comparison of peripancreatic exudate volume difference between EN and non-EN treatment in patients with local complications (× 10^4^ mm^3^).

RAC	APFC (*n* = 230)			ANC (*n* = 98)		
	EN group	Non-EN group	*P*-value	EN group	Non-EN group	*P*-value
MSAP	*n* = 53	*n* = 97	< 0.001	*n* = 17	*n* = 31	0.046
	−17.100 (−32.490∼−1.907)	3.440 (−4.210∼13.125)		−12.280 (−23.485∼3.690)	1.470 (−7.190∼8.190)	
SAP	*n* = 42	*n* = 38	< 0.001	*n* = 41	*n* = 9	0.008
	−6.565 (−26.753∼8.105)	10.920 (1.525∼26.390)		−14.620 (−52.650∼0.125)	9.920 (−10.480∼43.595)	
MSAP + SAP	*n* = 95	*n* = 135	< 0.001	*n* = 58	*n* = 40	< 0.001
	−11.530 (−29.260∼−0.440)	4.270 (−3.060∼17.200)		−13.205 (−33.120∼2.273)	1.540 (−7.693∼12.545)	

EN, enteral nutrition; non-EN, non-enteral nutrition. MSAP, moderately severe acute pancreatitis; RAC, the Revised Atlanta Classification; SAP, severe acute pancreatitis. APFC, acute peripancreatic fluid collection; ANC, acute necrotic collection.

**TABLE 4:** Comparison of peripancreatic exudate difference at different CT re-examination interval times in patients with or without EN with local complications (× 10^4^ mm^3^).

RAC	Re-examination interval	APFC			ANC		
		EN group	Non-EN group	*P*-value	EN group	Non-EN group	*P*-value
MSAP	≤ 7 days	*n* = 16	*n* = 65	< 0.001	*n* = 4	*n* = 10	0.572
		−14.420 (−33.040∼−1.133)	3.440 (−4.440∼14.760)	–	−3.775 (−10.615∼1.670)	−6.935 (−14.108∼8.405)	–
	8–15 days	*n* = 37	*n* = 32	< 0.001	*n* = 13	*n* = 21	0.049
		−17.450 (−33.205∼−2.517)	3.160 (−3.323∼10.100)	–	−15.980 (−24.040∼6.280)	1.940 (−1.555∼10.450)	–
SAP	≤ 7 days	*n* = 9	*n* = 19	0.013	*n* = 9	*n* = 4	0.217
		−8.09 (−25.275∼7.140)	10.950 (0.150∼23.250)	–	2.670 (−28.68∼9.710)	14.205 (−5.493∼24.370)	–
	8–15 days	*n* = 33	*n* = 19	0.001	*n* = 32	*n* = 5	0.046
		−2.31 (−27.520∼8.710)	10.890 (2.230∼31.190)	–	−24.400 (−55.503∼−0.895)	−4.330 (−12.295∼67.915)	–
MSAP + SAP	≤ 7 days	*n* = 25	*n* = 84	< 0.001	*n* = 13	*n* = 14	0.961
		−13.480 (−28.085∼−1.335)	4.370 (−3.279∼16.793)	–	−3.000 (−9.465∼6.140)	−6.375 (−11.498∼13.443)	–
	8–15 days	*n* = 70	*n* = 51	< 0.001	*n* = 45	*n* = 26	< 0.001
		−10.750 (−30.073∼−0.835)	4.270 (0.250∼17.200)	–	−20.580 (−42.485∼−0.405)	1.775 (−4.513∼12.820)	–

EN, enteral nutrition; non-EN, non-enteral nutrition. MSAP, moderately severe acute pancreatitis; RAC, the Revised Atlanta Classification; SAP, severe acute pancreatitis. APFC, acute peripancreatic fluid collection; ANC, acute necrotic collection.

### 3.3 Follow-up findings in patients

**FIGURE 3:**
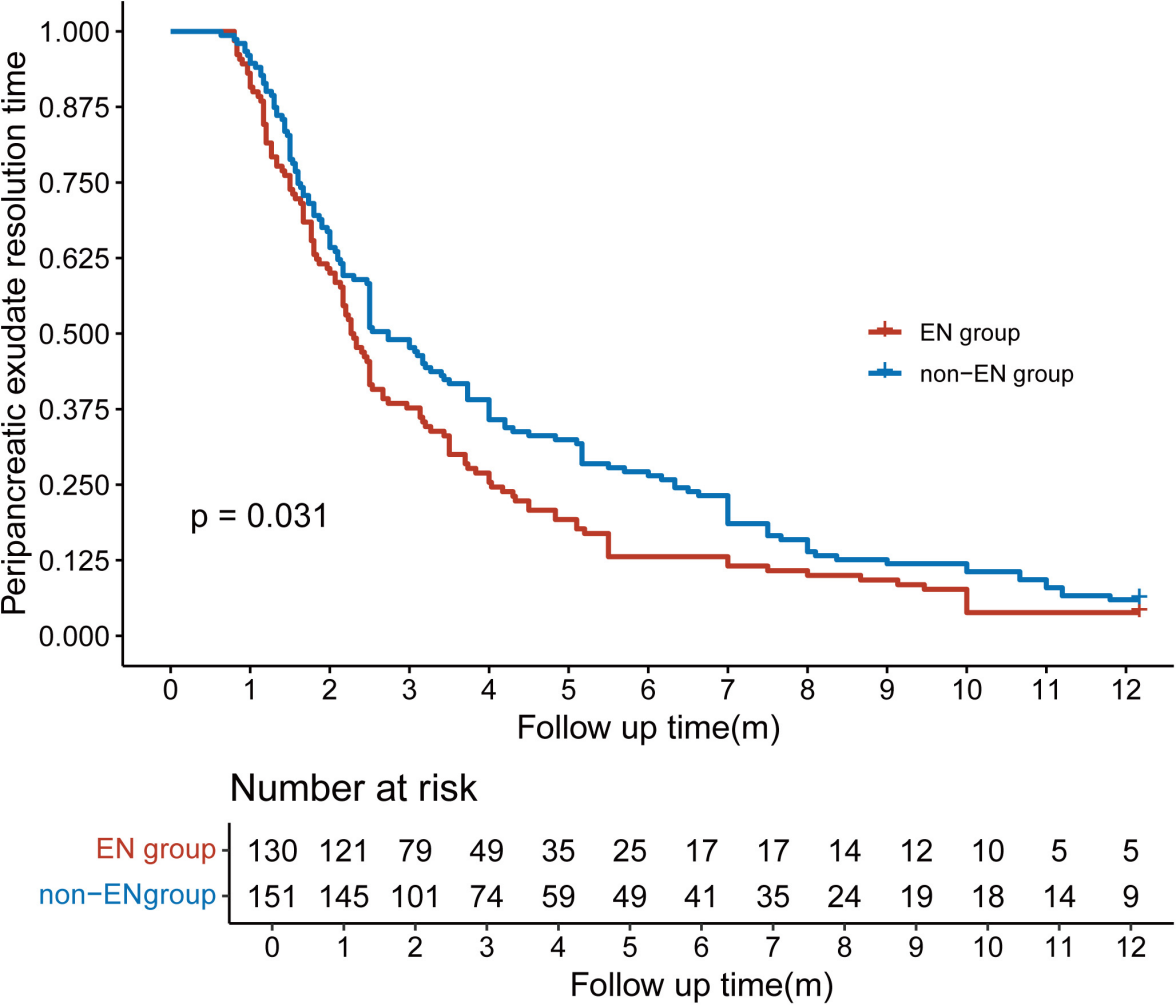
Comparison of time to complete resolution of peripancreatic exudate between the enteral nutrition (EN) and non-enteral nutrition (non-EN) groups.

In this study, all patients were followed up for 1 year. The Kaplan-Meier method was employed to construct the curve depicting the regression of peripancreatic exudate, thereby evaluating the effect of different treatment methods on peripancreatic exudate resolution. As shown in Figure 3, the mean time of complete resolution of peripancreatic exudate was 2.28 months in the EN group and 2.73 months in the non-EN group. The overall resolution time for patients in the EN group was shorter than that in the non-EN group (*p* = 0.031). The risk table demonstrates that the number of patients at risk in both groups progressively decreased over time. Specifically, 23 patients in the EN group and 24 patients in the non-EN group did not undergo assessment within the 1 year follow-up period. At the end of the 1 year follow-up, peripancreatic exudate had not resolved in five patients in the EN group and nine patients in the non-EN group, which mainly manifested as pancreatic pseudocysts and walled-off necrosis.

## 4 Discussion

AP is a common digestive disorder, is one of the most common diseases of the pancreas, and leads to significant short- and long-term morbidity (20–22). Following the initial episode, up to 40% of patients may develop new-onset prediabetes or diabetes. Additionally, approximately 25% of patients experience exocrine pancreatic insufficiency (23, 24). Furthermore, recurrence of AP occurs in approximately 18% of cases, and 8% of patients progress to chronic pancreatitis. Both recurrence and chronic progression impose additional financial burdens on the healthcare system (25, 26). Considering these challenges, it is an urgent need to design effective therapeutic agents for the therapy or prevention of AP. Clinical trials that have marked milestones have provided important perspectives on clinical management, including fluid resuscitation and nutritional support (27).

In AP, injury to pancreatic acinar and ductal cells leads to the release of inflammatory mediators, cellular damage, and increase in capillary permeability, which collectively induce capillary leak syndrome. This results in the extravasation of pancreatic enzymes, albumin, inflammatory mediators, and other substances from the vasculature into the third space. During the pathological process, activated pancreatic enzymes and cytokines may enter the peritoneal cavity, causing chemical burns and third-spacing of fluid. They can also spread through anatomical spaces, leading to extensive exudation. The leakage of pancreatic fluids can corrode surrounding tissues, resulting in peripancreatic tissue necrosis, which increases the complexity of treatment and prolongs hospitalization.

According to the 2012 revised classification of AP, MSAP, and SAP patients may develop local complications in the advanced stage of the disease, with the most common being APFC and ANC. It is important to evaluate local complications through imaging, as they may directly influence therapy strategies (17). Multiple RCT studies have shown that EN therapy can reduce the risk of infection, mortality, MODS, and duration of hospitalization in patients with SAP (13, 28–30). At present, most of the research on EN has focused on exploring its therapeutic impact on the above serious outcomes, which is a major concern in the field. However, there are few studies on the efficacy of treatment for local complications. For patients with MSAP and SAP, symptom alleviation and normalization of relevant blood test indicators after standardized clinical treatment do not necessarily indicate complete resolution of local complications. Therefore, it is essential to regularly evaluate the recovery of these local complications even after discharge. Currently, MCTSI is widely used for this purpose. However, the MCTSI has certain limitations. Specifically, it cannot quantify the volume of pancreatic or peripancreatic fluid collection or peripancreatic fat necrosis. Moreover, it fails to distinguish the severity of pancreatitis within the same score grade. These shortcomings may lead to inaccurate assessments of the patient’s condition in clinical practice, which in turn can affect treatment decisions and patient prognosis.

This study employed a method of delineating the pancreas and peripancreatic exudate on CT images to quantify the volume of peripancreatic exudate for the first time. By comparing the changes in peripancreatic exudate volume between patients treated with and without EN, we evaluated the impact of EN on local complications in patients with MSAP and SAP. This approach further substantiates the therapeutic benefits of EN in the management of AP.

The study revealed that after EN therapy, peripancreatic exudate significantly decreased. In contrast, in patients who did not receive EN therapy, peripancreatic exudate increased compared with that at the time of onset. As AP progresses, peripancreatic exudate tends to increase gradually. However, the use of EN can reduce peripancreatic exudate. In patients with APFC and ANC, the peripancreatic exudate volume was significantly reduced following EN therapy compared with that in patients who did not receive EN. This suggests that EN therapy can effectively alleviate local complications.

The optimal timing for initiating EN in AP remains controversial and is a major focus of clinical research. However, studies on the timing of EN therapy are currently lacking. In this study, we found that in patients with MSAP and SAP complicated with APFC, the peripancreatic exudate volume was significantly lower in those receiving EN compared with those without EN, at re-examination CT intervals of ≤ 7 days and 8–15 days. This indicates that EN can reduce peripancreatic fluid exudate in APFC patients within 1 week of initiation. In patients with MSAP and SAP complicated by ANC, the peripancreatic exudate volume was significantly lower in those receiving EN compared with the non-EN group at a re-examination CT interval of 8–15 days. No significant change was observed in patients with a re-examination interval of ≤ 7 days. Several factors likely explain this finding. First, an ANC comprises variable amounts of fluid and necrotic tissue (17). Clearance of this necrotic material depends on the slow and inefficient phagocytic activity of macrophages and other immune cells, a process that can last weeks to months. Second, during the acute phase, necrotic acinar cells continuously release abundant damage-associated molecular patterns (DAMPs), pro-inflammatory cytokines, and vasoactive mediators, generating a dominant local pro-inflammatory milieu (27). Consequently, although EN reduces infectious complications and modulates systemic immune responses, its anti-inflammatory and immunoregulatory effects are initially overwhelmed by this intense local inflammation. Finally, the small sample size in this subgroup may have limited our ability to detect subtle changes. Larger prospective studies are needed to confirm these observations.

In a 1 year follow-up study, we observed that patients receiving EN therapy had a significant advantage in the absolution of peripancreatic exudate. Specifically, the time to complete resolution of peripancreatic exudate was significantly shorter in the EN group compared with the non-EN group, indicating that EN therapy can more effectively promote the absolution of peripancreatic exudate.

The present study has the following limitations. First, this study only assessed the effects of EN on APFC and ANC, without evaluating the impact on local complications associated with pancreatic pseudocyst and walled-off necrosis. Future studies should further investigate the effects of EN on local complications related to pancreatic pseudocyst and walled-off necrosis to provide a more comprehensive understanding of the role of EN in local complications. Second, the retrospective design of this study may introduce selection bias and confounding factors. Future prospective studies with better control of confounding factors are needed to more accurately evaluate the effects of EN on local complications in patients with MSAP and SAP.

In conclusion, this study underscores the therapeutic advantages of EN in the therapy of AP, particularly in reducing peripancreatic exudate and alleviating local complications. Our findings support the integration of EN as a cornerstone of clinical management strategies for AP. Future research should continue to explore the mechanisms underlying the benefits of EN and identify optimal protocols for its implementation in clinical practice.

## Data Availability

The original contributions presented in this study are included in this article/Supplementary material, further inquiries can be directed to the corresponding authors.
