# Supplementary material for: Assessing the impact of enteral nutrition on peripancreatic exudate in acute pancreatitis using CT volume measurement
Source: Front Med (Lausanne). 2025 Sep 18;12:1660818. doi: 10.3389/fmed.2025.1660818 (PMC12488576; doi:10.3389/fmed.2025.1660818)
Supplement: Supplementary file 1 [file Data_Sheet_1.pdf]

Assessing the Impact of Enteral Nutrition on Peripancreatic Exudate in Acute Pancreatitis Using CT Volume Measurement

Contents

sTable 1 Baseline Characteristics of Patient with MSAP
sTable 2 Baseline Characteristics of Patient with SAP

sTable 1 Baseline Characteristics of Patient with MSAP

**Characteristic**	**EN group (n=70)**	**non-EN group (n=128)**	***P* value[a]**
**Gender**			0.515
Male	41(58.57)	81(63.28)	
Female	29(41.43)	47(36.72)	
**Age，y**			0.089
Median (IQR)	42(31.75-56)	47.5(33-62.75)	
**BMI, kg/m^2**	25.721±3.167	25.238±3.784	0.365
**Smoking history**			0.699
Yes	13(18.57)	21(16.41)	
No	57(81.43)	107(83.59)	
**Alcohol consumption history**			0.100
Yes	17(24.29)	19(14.84)	
No	53(75.71)	109(85.16)	
**history of diabetes mellitus**			0.870
Yes	11(15.71)	19(14.84)	
No	59(84.29)	109(85.16)	
**Etiology of AP**			0.647
Gallstones	27(38.57)	55(42.97)	
Hypertriglyceridaemia	31(44.29)	48(37.50)	
Other[b]	12(17.14)	25(19.53)	
**SIRS at admission**			0.904
Yes	40(57.14)	72(56.25)	
No	30(42.86)	56(43.75)	
**BISAP within 24 hours after admission**			0.434
Median (IQR)	2(1-2)	2(1-2)	
**MCTSI score**			0.434
Median (IQR)	4(4-6)	4(4-6)	
**Length of stay, d**			0.066
Median (IQR)	13.5(10-16)	12(9-15)	

EN, enteral nutrition; non-EN, non-enteral nutrition. RAC, the Revised Atlanta Classification.

a: Students's t-test, Pearson Chi-squared test, Mann-Whitney U test.

b: alcoholic, post-ERCP, malignant tumor, intraductal papillary mucinous neoplasm and idiopathic pancreatitis.

sTable 2 Baseline Characteristics of Patient with SAP

Characteristic	EN group (n=83)	non-EN group (n=47)	*P* value[a]
**Gender**			0.573
Male	47(56.63)	29(61.70)	
Female	36(43.37)	18(38.30)	
**Age，y**			0.606
Median (IQR)	47(35-61)	49(35-68)	
**BMI, kg/m^2**	26.184±4.555	26.004±4.769	0.832
**Smoking history**			0.982
Yes	14(16.87)	8(17.02)	
No	69(83.13)	39(82.98)	
**Alcohol consumption history**			0.696
Yes	22(26.51)	11(23.40)	
No	61(73.49)	36(76.60)	
**history of diabetes mellitus**			0.750
Yes	16(19.28)	8(17.02)	
No	67(80.72)	39(82.98)	
**Etiology of AP**			0.313
Gallstones	36(43.37)	21(44.68)	
Hypertriglyceridaemia	32(38.56)	13(27.66)	
Other[b]	15(18.07)	13(27.66)	
**SIRS at admission**			0.074
Yes	65(78.31)	30(63.83)	
No	18(21.69)	17(26.17)	
**BISAP within 24 hours after admission**			0.164
Median (IQR)	2(2-3)	2(2-3)	
**MCTSI score**			0.252
Median (IQR)	8(6-8)	8(6-8)	
**Length of stay, d**			0.689
Median (IQR)	19(15-25)	18(13-25)	

EN, enteral nutrition; non-EN, non-enteral nutrition. RAC, the Revised Atlanta Classification.

a: Students's t-test, Pearson Chi-squared test, Mann-Whitney U test.

b: alcoholic, post-ERCP, malignant tumor, intraductal papillary mucinous neoplasm and idiopathic pancreatitis.
